# Evolutionary selection identifies critical immune-relevant genes in lung cancer subtypes

**DOI:** 10.3389/fgene.2022.921447

**Published:** 2022-08-24

**Authors:** Kimberly A. Luddy, Jamie K. Teer, Audrey Freischel, Cliona O’Farrelly, Robert Gatenby

**Affiliations:** ^1^ Cancer Biology and Evolution Program, H. Lee Moffitt Cancer Center and Research Institute, Tampa, FL, United States; ^2^ Integrated Mathematical Oncology, H. Lee Moffitt Cancer Center and Research Institute, Tampa, FL, United States; ^3^ Biostatistics and Bioinformatics, H. Lee Moffitt Cancer Center and Research Institute, Tampa, FL, United States; ^4^ School of Biochemistry and Immunology, Trinity College Dublin, Trinity Biomedical Sciences Institute, Dublin, Ireland; ^5^ School of Medicine, Trinity College Dublin, Dublin, Ireland

**Keywords:** cancer evolution, cancer immunology, evolutionary triage, gene conservation, immune evasion

## Abstract

In an evolving population, proliferation is dependent on fitness so that a numerically dominant population typically possesses the most well adapted phenotype. In contrast, the evolutionary “losers” typically disappear from the population so that their genetic record is lost. Historically, cancer research has focused on observed genetic mutations in the dominant tumor cell populations which presumably increase fitness. Negative selection, i.e., removal of deleterious mutations from a population, is not observable but can provide critical information regarding genes involved in essential cellular processes. Similar to immunoediting, “evolutionary triage” eliminates mutations in tumor cells that increase susceptibility to the host immune response while mutations that shield them from immune attack increase proliferation and are readily observable (e.g., B2M mutations). These dynamics permit an “inverse problem” analysis linking the fitness consequences of a mutation to its prevalence in a tumor cohort. This is evident in “driver mutations” but, equally important, can identify essential genes in which mutations are seen significantly less than expected by chance. Here we utilized this new approach to investigate evolutionary triage in immune-related genes from TCGA lung adenocarcinoma cohorts. Negative selection differs between the two cohorts and is observed in endoplasmic reticulum aminopeptidase genes, ERAP1 and ERAP2 genes, and DNAM-1/TIGIT ligands. Targeting genes or molecular pathways under positive or negative evolutionary selection may permit new treatment options and increase the efficacy of current immunotherapy.

## Introduction

Emergence of a clinical cancer population is typically a prolonged Darwinian process in which the malignant cells evolve phenotypic properties that adapt to tissue growth constraints and host immune responses. These evolutionary dynamics occur at the level of phenotypic interactions with environmental selection forces and accumulating genomic changes provide a record of this evolutionary arc. For example, during immunoediting, mutations that protect tumor cells from the immune system enhance proliferation and, therefore, have increased prevalence in the population. In contrast, mutations that increase recognition by the immune system, because they decrease fitness and proliferation, are typically lost from the population. Current molecular biology techniques prioritize the study of “driver” mutations that are observed within some large fraction of the tumor population. These provide important insights into critical pathways and potential therapeutic targets. However, prior theoretical studies have found identifying mutations subjected to negative selection can be equally informative. However, lost mutations cannot be measured directly in a tumor sample and require novel approaches.

**FIGURE 1:**
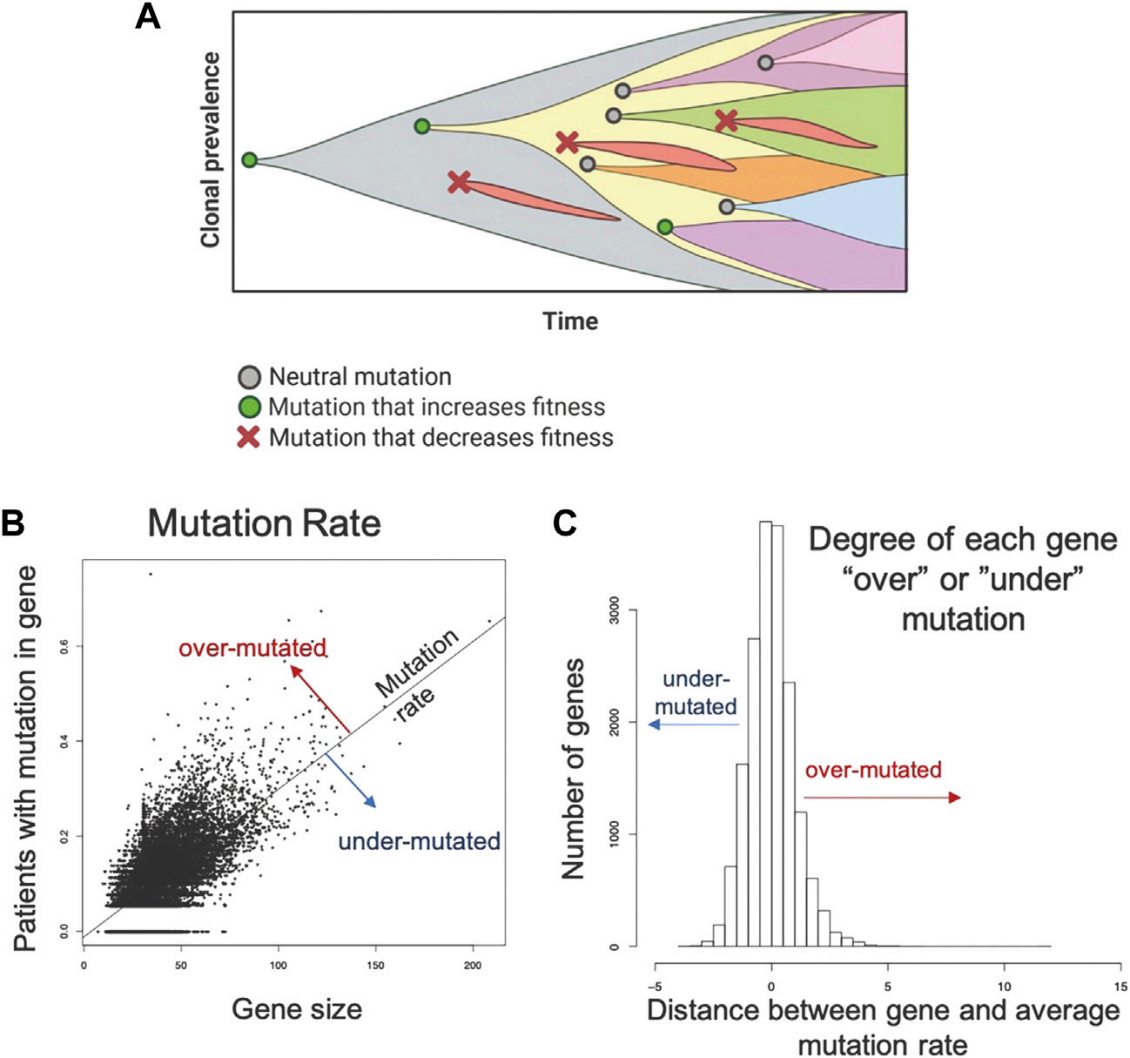
Methodology applied to the TCGA data set to identify genes that are mutated more or less frequently than expected by chance alone. **(A)** Evolution is a process of neutral (grey circle), positive (green circle), and negative selection (red x-mark) events resulting in the accumulation of mutations that increase cell fitness and removal of mutations that decrease cell fitness. **(B)**. A robust regression model was used to establish the mutation rate based on the fraction of patients with a protein altering mutation in each gene against the size of the gene. Distance from the line for each gene was used to quantify conserved and hypermutated genes. Genes on the regression line have background levels of mutations. **(C)** Distance from the regression model for each gene. Genes with an SR ≥ 1.0 are hypermutated and genes with an SR ≤ -1.0 are conserved genes (under mutated). All others have an expected number of mutations (Background mutations).

In prior studies, the principle of “evolutionary triage (Gatenby et al., 2014)”, which links the prevalence of any gene mutation in a population with its contribution to cancer cell fitness (Gatenby and Frieden, 2002; Gatenby and Frieden, 2004). A mutation that increases fitness will also increase proliferation so that it is observed more frequently either within a tumor population or in a cohort of patients with that tumor. A mutation that does not alter fitness will not change proliferation and, therefore, be observed with a frequency that reflects the underlying mutation rate (Kimura, 1968; Gatenby and Frieden, 2002; Gatenby and Frieden, 2004). These represent the well-recognized dynamics of “driver” and “passenger” mutations. Computer simulations also demonstrated some genes must continue to function normally for optimal cancer cell fitness. In these genes, non-synonymous mutations cannot improve function and, so, will frequently decrease fitness and disappear from the population. These “essential” genes will usually have an observed prevalence of mutations that is less than expected by chance alone (i.e., less frequently than a passenger gene of comparable size) (Figure 1A)**.**

The dynamics of negative selection are well recognized, but prior investigations across multiple cancer types found few genes are broadly conserved (Martincorena et al., 2018; Zapata et al., 2018). However, computer simulations demonstrated evolutionary conservation in cancer populations varies depending on local tissue environment selection forces and prior molecular changes in their evolutionary arc (Gatenby et al., 2014; Cunningham et al., 2015). Thus, genes conserved among cancers emerging from different tissue types and/or through different initiating driver genes are predicted to be uncommon. Recent studies of TCGA data in *EGFR*-mut, *KRAS*-mut, and wild-type (no known driver mutations) (WT) lung adenocarcinomas, confirmed this prediction as few genes were found to be conserved even among subtypes that shared a common tissue of origin (Freischel et al., 2021).

Here we investigate evolutionary triage in genes associated with tumor-immune interactions. Epithelial cells are active participants in the host immune response (Schleimer et al., 2007). Antigen presentation, immune-modulating protein expression (e.g., checkpoint ligands), as well as cytokine and chemokine secretion by epithelial cells can promote or reduce host immune functions (Schleimer et al., 2007; Larsen et al., 2020). Additionally, epithelial cells express surface and cytoplasmic pattern recognition receptors (PRR), which recognize molecular patterns derived from foreign pathogens and markers of stressed cells triggering intrinsic immune responses within epithelial cells, including release of inflammatory mediators and initiation of pyroptosis (Amarante-Mendes et al., 2018).

We hypothesized that, within these diverse and complex interactions, we could determine the most important cellular functions and molecular pathways by identifying genes under strong positive and negative evolutionary selection. Furthermore, by investigating cancers arising from the same organ but with different initiating oncogenic mutations, we could investigate divergence in immune-evasion strategies arising from different initiating genetic events.

Here we explore this hypothesis by investigating the mutational frequency of genes with known or expected functions in host-immune interactions in two cohorts of lung adenocarcinoma patients in the TCGA database, mutant KRAS (*n* = 163) and no known driver (WT) (*n* = 313). Evolutionary selection of multiple genes with well-documented roles in LUAD supports our hypothesis and encourages further investigation of other evolutionarily identified genes and molecular pathways that have not been extensively investigated.

## Methods

### Data collection

Mutations detected by the Multi-Center Mutation Calling in Multiple Cancers (MC3) project on TCGA lung adenocarcinoma samples were downloaded from the Genome Data commons (file: mc3.v0.2.8.PUBLIC.maf.gz, site: https://gdc.cancer.gov/about-data/publications/mc3-2017). Details of MC3 mutation identification using tumor and matched normal samples are provided here: [PMC6075717].

We identified one sample each from patients belonging to the lung adenocarcinoma cohort, and classified patients based on known driver or recurrent mutations in *KRAS* (G12, G13, Q61, A146)*, BRAF* (V600, N581, G464, G466, G469, G596, D594)*,* and *EGFR* (L858, S768, L861, G719, T790, indels in exons 18–21). Samples were excluded if they matched criteria for more than one of these genes. Samples that did not meet any of the mutation criteria were classified as WT (no driver mutations in *EGFR, KRAS,* or *BRAF*.) Cohorts with *EGFR*-mutant LUAD (n = 58) and *BRAF*-mutant LUAD (n = 31) had fewer samples, so we elected to focus primarily on the *mKRAS* (n = 163) and WT (n = 313) cohorts. Tumor and normal sequence alignment files (BAM) were downloaded from the Genome Data Commons, and gene-level depth of coverage was calculated by calculating bases covered by sequencing from the above files across each of the RefSeq coding genes (with 25 base pair flanking regions). A base was considered sufficiently covered if the depth of coverage was≥14 in tumor sample and≥8 in normal samples (as has been previously described: https://www.synapse.org/#!Synapse:syn1695394). The fraction of each gene’s protein coding bases (using the longest RefSeq transcript) covered by sufficient sequence data was calculated for each sample using the Negative Storage Model [PMC7157186].

### Identification of over and under-mutated genes

To identify over- and under-mutated genes, we calculated the fraction of patients in each cohort with any protein-altering (nonsynonymous, truncating, canonical splice-site) mutations in each gene. To control for undercounting of mutations due to poor coverage, we divided the fraction patients mutated by the median fraction of coding bases with sufficient coverage. Genes with median coverage <50%, ANNOVAR annotation errors, or gene expression <2.0 log2 counts were excluded, as these genes either have artificially low mutation rates or are not likely to be essential due to low expression. Gene size was controlled by calculating a linear model between corrected mutation fraction and gene size (protein coding bases from largest RefSeq transcript.) (Figure 1B). Corrected mutation rate and gene size were both square-root transformed. The standardized residual of each gene to the regression line was determined: the most positive genes are the classical over-mutated or driver genes, and in each cohort the most highly mutated gene had the highest standardized residual (e.g., *KRAS* was the most over-mutated gene in the m*KRAS* cohort.) Genes with an SR ≥ 1.0 are over-mutated and genes with and SR ≤ -1.0 are conserved genes (Figure 1C). To minimize false positives, our analysis primarily focuses on genes with standardized residual (SR) values for conserved genes SR ≤ -2.0 to achieve *p* = 0.05 and SR ≤ -1.65 to achieve *p* = 0.1.

### Curated gene lists

**FIGURE 2:**
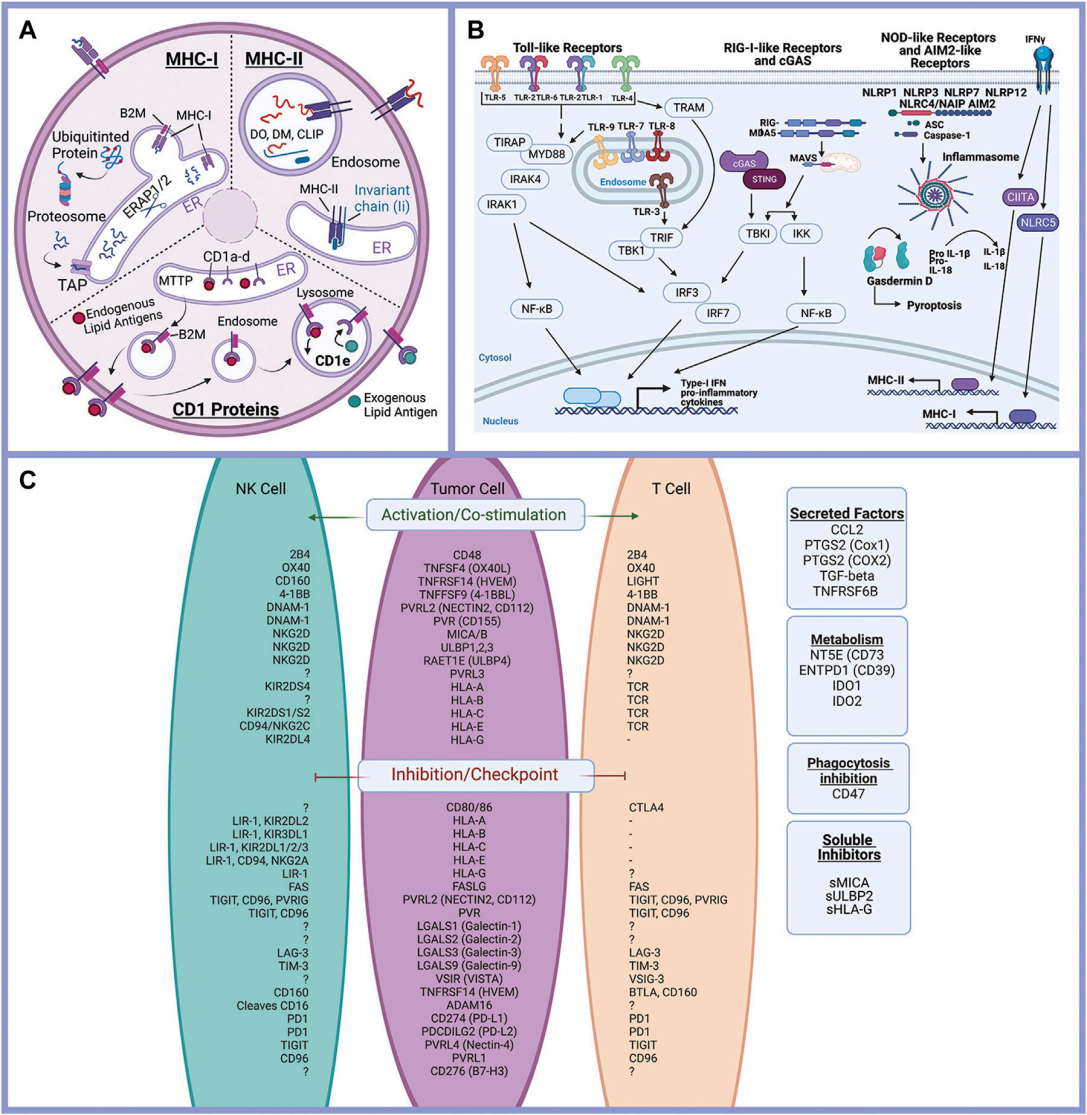
Epithelial cells and the immune response. Antigen processing and presentation of peptide antigens and lipid antigens occurs in malignant epithelial cells and alters the anti-tumor immune response **(A)**. Innate immune receptor signaling is triggered by a variety of ligands triggering expression of inflammatory mediators and activation of pyroptosis (programed cell death) **(B)**. Immune modulating proteins including checkpoint ligands, co-stimulatory molecules, and suppressive tumor cells regulate the anti-tumor immune response **(C)**. Created with Biorender.com.

Literature review and gene databases including Gene Set Enrichment (Broad Institute) (GSEA) (Subramanian et al., 2005), PathCards (Belinky et al., 2015), and Genenames.org (Tweedie et al., 2021) were used to create lists of genes with known or suspected functions in epithelial cell interactions with the immune response. Genes were divided into eight functional processes: antigen presentation (Figure 2A), immune-modulating proteins (checkpoint ligands) (Figure 2C), innate immune receptor signaling (Figure 2B), cytokine signaling, chemokine signaling, Interferon signaling, complement, and programmed cell death (apoptosis, necrosis, and pyroptosis)**.** Complete gene lists are provided (Supplementary Table S1).

## Results

### Gene conservation differs between the wild-type and mutant KRAS cohorts

More genes were highly conserved (SR ≤ ^-^2.0) in mKRAS than WT cancers (160 and 248 respectively) (Supplementary Table S2). Among the immune genes examined (n = 576) 19 were conserved (SR ≤ -1.65) in WT cohort and 11 in mKRAS (Table 1). Many of the conserved genes were unique to each cohort with only one overlapping pair of genes. Complement genes *C4A* (WT SR = -2.863, mKRAS SR = -1.711) and C4B (WT SR = -2.864, mKRAS SR = -1.711), which encode the acidic and basic forms of complement factor 4, an activator of the classical complement pathway, were conserved in both cohorts. No genes from the list of chemokines and chemokine receptors were highly conserved in either cohort (Supplementary Figures S1A,B).

**TABLE 1:** Highly conserved genes in wild-type (top) and mKRAS (bottom) tumors. SR ≤ -1.65.

Function	Gene symbol	Gene name	SR value wild-type
Immune Modulating Proteins	PVRL2	Nectin Cell Adhesion Molecule 2	−2.054
	PVR	PVR Cell Adhesion Molecule	−1.782
	MICA	MHC Class I Polypeptide-Related Sequence A	−1.699
Antigen Processing	ERAP1	Endoplasmic Reticulum Aminopeptidase 1	−2.797
	TAP1	Transporter 1, ATP Binding Cassette Subfamily B Member	−2.566
	HLA-F	Major Histocompatibility Complex, Class I, F	−1.841
Innate Immune Receptor Signaling	IRF3	Interferon Regulatory Factor 3	−1.865
Interferon Signaling	IRF2BP1	Interferon Regulatory Factor 2 Binding Protein 1	−2.149
	SOCS6	Suppressor Of Cytokine Signaling 6	−2.048
	IRF5	Interferon Regulatory Factor 5	−2.003
	IRF3	Interferon Regulatory Factor 3	−1.865
	SIRT3	Sirtuin 3	−1.739
Cytokine Signaling	IL1R1	Interleukin 1 Receptor Type 1	−2.118
	IL17RB	Interleukin 17 Receptor B	−1.977
	TNFRSF10A	TNF Receptor Superfamily Member 10a	−1.901
	TNFRSF1A	TNF Receptor Superfamily Member 1A	−1.871
	TGFB3	Transforming Growth Factor Beta 3	−1.770
	IL6ST	Interleukin 6 Cytokine Family Signal Transducer	−1.753
Apoptosis/necrosis/Pyroptosis	TNFRSF10A	TNF Receptor Superfamily Member 10a	-1.901
Complement	C4B	Complement C4B (Chido Blood Group)	−2.864
	C4A	Complement C4A (Rodgers Blood Group)	−2.863
**Function**	**Gene Symbol**	**Gene Name**	**SR value mKRAS**
Antigen Processing	CIITA	Class II Major Histocompatibility Complex Transactivator	−2.296
	ERAP2	Endoplasmic Reticulum Aminopeptidase 2	−2.085
Innate Immune Receptor Signaling	CIITA	Class II Major Histocompatibility Complex Transactivator	−2.296
	NLRC3	NLR Family CARD Domain Containing 3	−2.217
	DHX58	DExH-Box Helicase 58	−1.687
Interferon Signaling	SIRT1	Sirtuin 1	−1.791
Cytokine Signaling	LTBP3	Latent Transforming Growth Factor Beta Binding Protein 3	−2.496
	IL17RD	Interleukin 17 Receptor D	−1.779
	TGFBI	Transforming Growth Factor Beta Induced	−1.694
Apoptosis/necrosis/Pyroptosis	CASP8AP2	Caspase 8 Associated Protein 2	−3.160
Complement	C4B	Complement C4B (Chido Blood Group)	−1.711
	C4A	Complement C4A (Rodgers Blood Group)	−1.711

#### Differential conservation in antigen processing genes

**FIGURE 3:**
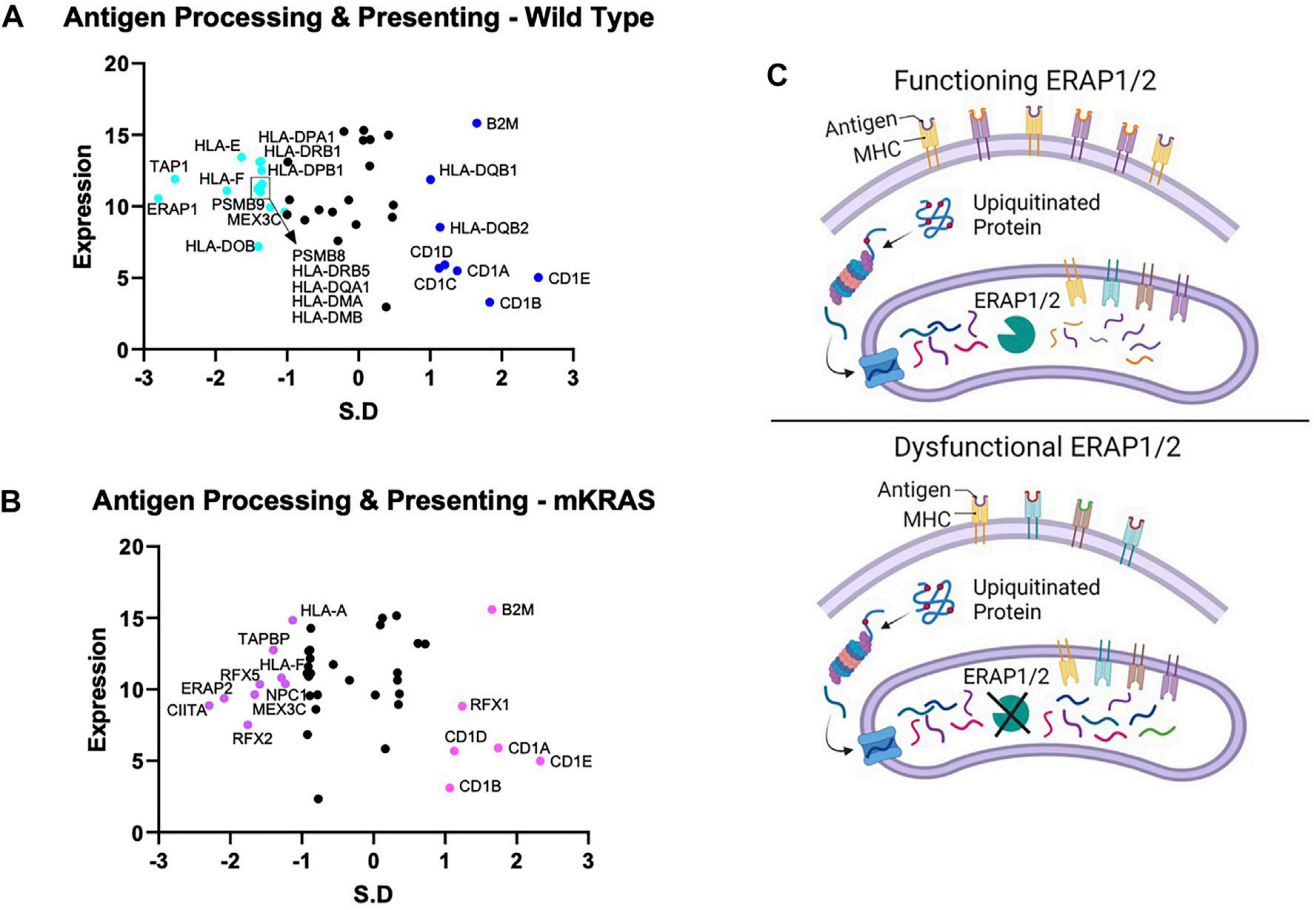
Gene conservation in antigen presentation genes. Gene conservation score, measured as the standard residual from the regression line (SR) graphed against genes expression levels (Log2) for wild-type (no identifiable driver mutations) (WT) **(A)** and mutant KRAS (mKRAS) **(B)**. Conserved genes (SR ≤ -1.0) and over-mutated genes (SR ≥ 1.0) are colored and labelled with text. ERAP1 and ERAP2 sculpt the peptide pool prior to presentation by MHC-I proteins. Dysfunctional ERAP alters the peptides presented **(C)**. Standardized residual (SR). Created with Biorender.com.

Endogenous proteins are degraded into antigen precursors in the cytoplasm by the (immuno)proteasome. Transporter associated with antigen processing (TAP) transports the protein fragments into the endoplasmic reticulum (ER) for further processing by aminopeptidases and loading onto MHC-I proteins. Dysregulation of this process alters the peptide pool presented and loss of integral component results in diminished MHC-I expression. Transporter 1, ATP Binding Cassette Subfamily B Member, TAP1 was conserved in the WT cohort (SR = -2.566) but not in the mKRAS group (SR = -0.561) (Figures 3A,B). TAP1 co-localizes with TAP2 and mediates the flow of peptides from the cytosol into the endoplasmic reticulum prior to cleavage by ERAP proteins. TAP2 gene expression and conservation score could not be determined from our dataset.

The complexity of evolutionary selection is notable in the endoplasmic reticulum aminopeptidase genes, *ERAP1* and *ERAP2,* which shape the peptidome by cleaving peptide precursors after transport into the ER (Figure 3C). *ERAP1* is among the most conserved genes in WT cancers (SR = -2.797) and *ERAP2* among the most conserved in mKRAS (SR = -2.085) (Figures 3A,B). Interestingly, no *ERAP1* mutations were found in any mEGFR tumors or comparable TCGA data from melanoma patients (data not shown), suggesting its function is essential in multiple cancer types.

#### Polio virus receptor gene conservation in wild-type tumors

**FIGURE 4:**
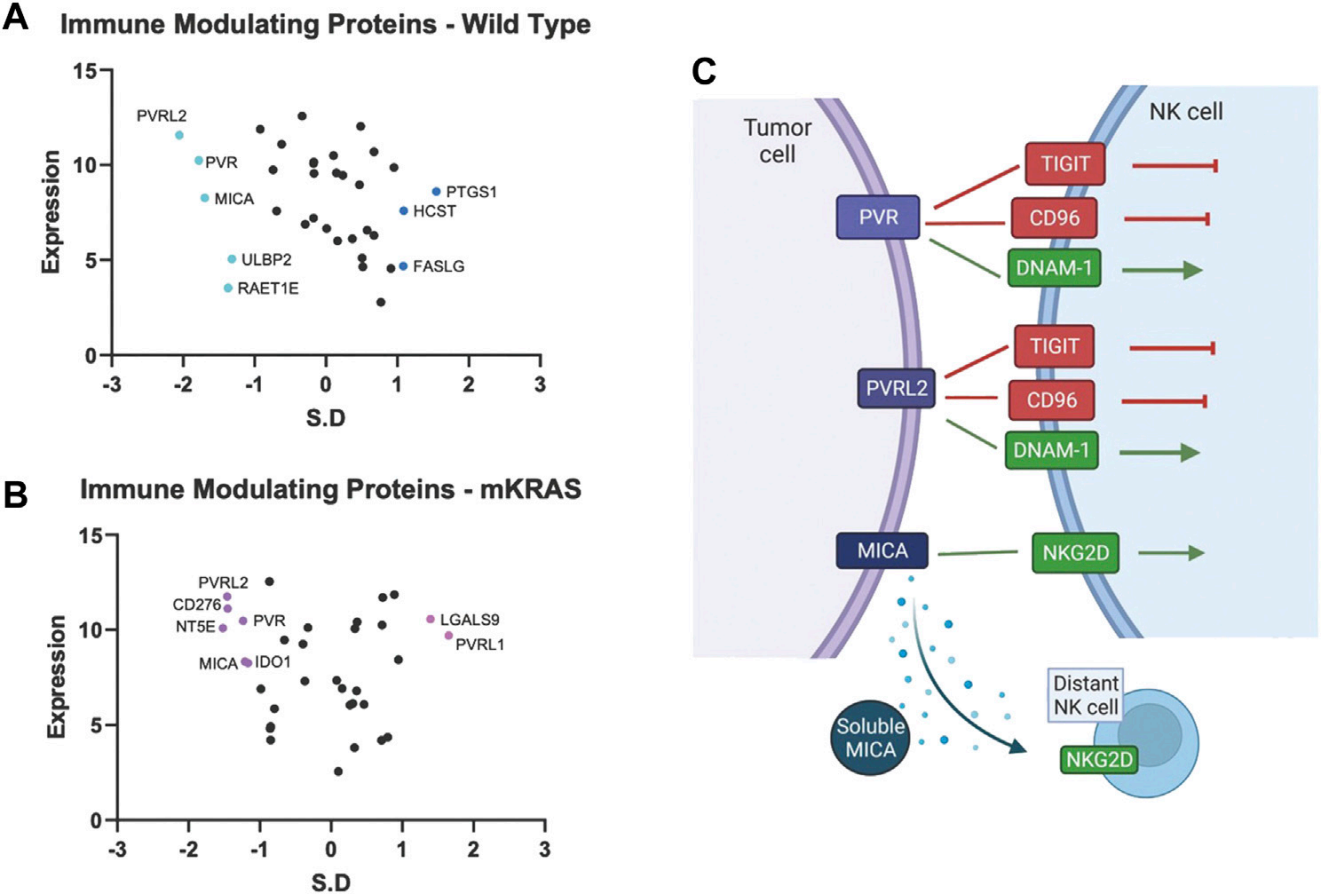
Gene conservation in genes encoding immune modulating proteins highlights the important role of NK cells in lung cancer. Gene conservation score, measured as the standard residual from the regression line (SR) graphed against genes expression levels (Log2) for WT **(A)** and mKRAS **(B)**. Conserved genes (SR ≤ -1.0) and over-mutated genes (SR ≥ 1.0) are colored and labelled with text. PVR, PVRL2, and MICA can activate or inhibit NK cells depending on NK receptor expression and solubility of MICA **(C)**. Standardized residual (SR). Created with Biorender.com.

Tumor cells express immune modulating proteins that activate or inactivate (checkpoint proteins) immune cells. None of the immune modulating proteins were significantly conserved in the mKRAS group. Polio Virus Like Receptor 2 (PVRL2, Nectin2, CD112) and PVR (CD155), conserved in WT (SR = -2.054 and -1.782, respectively), were also conserved in mKRAS cohort although did not reach significance (SR = -1.458 and -1.234, respectively) (Figures 4A,B). The conserved tumoral PVR proteins represent known tumor evasions strategies and can activate or inhibit (Wu et al., 2021) NK cells and T cells (Figure 4C).

MHC class I polypeptide–related sequence A (MICA) a stress-induced activating ligand for Natural Killer Cell Lectin Like Receptor, *KLRK1* (*NKG2D*) was conserved in the wild-type cohort (SR = -1.699). Ligation of *NKG2D* activates NK cells and gamma-delta-T cells and co-stimulates CD8 T cells (Holdenrieder et al., 2006). However, MICA can be cleaved from the cell surface and inactivate distant NK cells, NKT cells, gamma-delta T cells, and CD8 T cells (Uhlenbrock et al., 2014). Numerous proteases, including MMPs, ADAM10, and ADAM17, are involved in MICA cleavage. Additional NKG2D ligands, RAET1E (ULBP4) and ULBP2, were also conserved in the wild-type cohort but did not reach the SR cutoff of -1.65 (WT SR = -1.37 and -1.32, respectively).

#### Conservation of cytokine signaling genes

**FIGURE 5:**
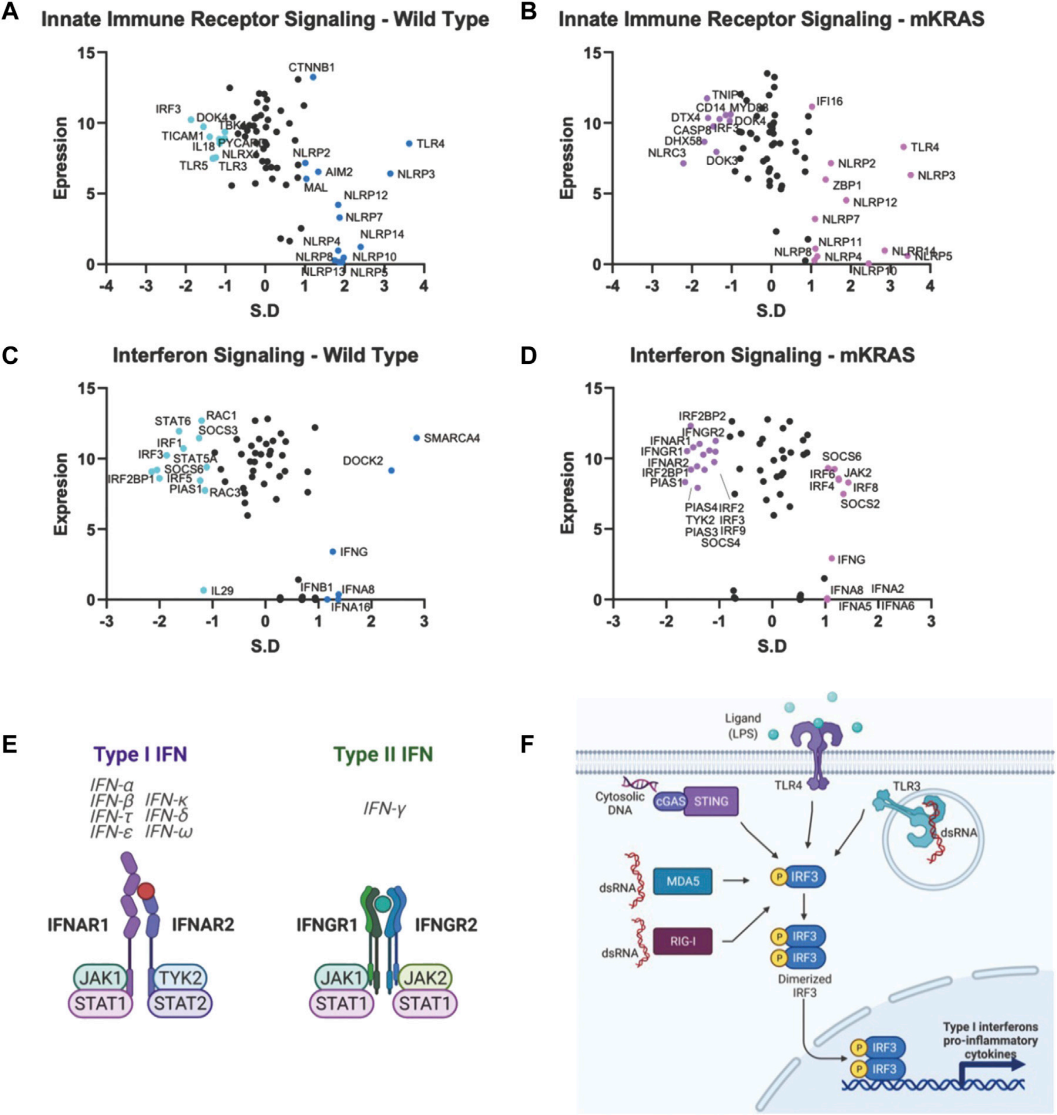
Gene conservation in pro-inflammatory genes. Gene conservation score, measured as the standard residual from the regression line (SR) graphed against genes expression levels (Log2). Innate immune signaling genes in WT **(A)** and mKRAS **(B)**. Interferon signaling genes in WT **(C)** and mKRAS **(D)**. Conserved genes (SR ≤ -1.0) and over-mutated genes (SR ≥ 1.0) are colored and labelled with text. Type-I and Type-II interferon receptors are conserved in mKRAS **(E)**. Interferon regulatory factor 3 (IRF3) is a central component in multiple pattern recognition receptor pathways **(F)**. Standardized residual (SR). Created with Biorender.com.

Malignant epithelial cells secrete and detect cytokines in the tumor microenvironment that regulate the immune response and support pro-growth signals. The potential for false positives is increased in genes with a conservation score above SR = -1.65. However, we note that both subunits of the type I IFN receptor, interferon-alpha receptor 1 (IFNAR1) and IFNAR2, are conserved in mKRAS samples (SR = -1.490 and SR = -1.418, respectively) (Figures 5D,E). Stimulation of IFNAR1/2 activates Janus kinase 1 (JAK1), (neutral in mKRAS) and tyrosine kinase 2 (TYK2) (mKRAS SR = -1.070). Type II interferon-gamma receptors IFNGR1 and INFGR2 were also conserved in mKRAS but did not meet the -1.65 threshold (SR = -1.371 and SR = -1.069, respectively) (Figures 5D,E). IFN-γ signaling in epithelial cells induces the expression of multiple genes containing γ-interferon activated sequences. MHC-I, checkpoint inhibitor PD-L1, and immunosuppressive enzyme, IDO are upregulated on cancer cells by IFN-γ treatment (21). All interferon receptor genes were neutral in the wild-type cohort.

Two of the nine interferon regulatory factor (IRF) genes were highly conserved and expressed in the wild-type cohort. IRF5, conserved in WT (SR = -2.002) and neutral in mKRAS, regulates type-I interferon genes and IFN stimulated genes (ISG) (Figures 5C,D). Similarly, activated IRF-3, conserved in WT (SR = -1.864) translocates to the nucleus following phosphorylation by TKB1 (WT SR = -1.010) and IKBKE (WT neutral) kinases and induces expression of type I interferons and ISGs. IRF3 is activated by a variety of pattern recognition receptors which are neutral in both cohorts (Figures 5A,B,F) (Zhu et al., 2010). Interleukin 1 receptor-1 (IL1R1) is the most conserved among the cytokine signalling genes in WT tumors (Supplementary Figure S1C)**.** Following ligation by either IL1A or IL1B, IL1R1 associates with interleukin-1 rector accessory protein (IL1RAP) (WT SR = -1.116) and activates many downstream proinflammatory pathways including NF-κB and MAPK (44).

### Over-mutated genes are shared between WT and mKRAS cohorts

Mutations can inhibit or enhance protein function. Positive selection occurs when the mutation increases fitness. WT and mKRAS tumors averaged 348 and 300 total mutations per tumor compared mEGFR LUADs with 86 (data not shown). Frequently mutated genes, defined as genes mutated in greater than 10% of patients in each cohort, numbered 208 and 157 in the WT and mKRAS cohorts, respectively. Genes with the highest mutation scores in our cohorts reflect known drivers and frequently mutated genes including KRAS, TP53, KEAP1, and STK11 (Supplementary Table S3) (Gong et al., 2020), demonstrating the validity of this approach. Of the immune related genes we examined (n = 576), 29 were over-mutated in WT (SR ≥ 1.65) and 26 in mKRAS with 16 genes common between the subtypes (Table 2).

**TABLE 2:** Over-mutated genes in WT (top) and mKRAS (bottom) cohorts. SR ≤ -1.65.

Function	Gene symbol	Gene name	SR value wild-type
Immune Modulating Proteins	CD86	CD86 Molecule	1.693
Antigen Processing	CD1E	CD1e Molecule	2.513
	CD1B	CD1b Molecule	1.831
	B2M	Beta-2-Microglobulin	1.653
Innate Immune Receptor Signaling	TLR4	Toll Like Receptor 4	3.626
	NLRP3	NLR Family Pyrin Domain Containing 3	3.150
	NLRP14	NLR Family Pyrin Domain Containing 14	2.404
	NLRP5	NLR Family Pyrin Domain Containing 5	1.975
	NLRP10	NLR Family Pyrin Domain Containing 10	1.936
	NLRP7	NLR Family Pyrin Domain Containing 7	1.874
	NLRP13	NLR Family Pyrin Domain Containing 13	1.865
	NLRP12	NLR Family Pyrin Domain Containing 12	1.838
	NLRP4	NLR Family Pyrin Domain Containing 4	1.836
	NLRP8	NLR Family Pyrin Domain Containing 8	1.753
Interferon Signaling	SMARCA4	SWI/SNF Related, Matrix Associated, Actin Dependent Regulator Of Chromatin, Subfamily A, Member 4	2.854
	DOCK2	Dedicator Of Cytokinesis 2	2.378
Cytokine Signaling	LTBP1	Latent Transforming Growth Factor Beta Binding Protein 1	2.766
	IL7R	Interleukin 7 Receptor	1.845
	IL18RAP	Interleukin 18 Receptor Accessory Protein	1.804
	IL1RAPL1	Interleukin 1 Receptor Accessory Protein Like 1	1.758
Apoptosis/necrosis/Pyroptosis	SERPINB4	Serpin Family B Member 4	2.140
Complement	CSMD3	CUB And Sushi Multiple Domains 3	6.194
	CSMD1	CUB And Sushi Multiple Domains 1	3.701
	C7	Complement C7	2.656
	ITGAX	Integrin Subunit Alpha X	2.277
	CSMD2	CUB And Sushi Multiple Domains 2	2.238
	CFH	Complement Factor H	1.940
	CFHR5	Complement Factor H Related 5	1.926
	C6	Complement C6	1.715
**Function**	**Gene Symbol**	**Gene Name**	**SR value mKRAS**
Immune Modulating Proteins	PVRL1	Nectin Cell Adhesion Molecule 1	1.653
Antigen Processing	CD1E	CD1e Molecule	2.329
	CD1A	CD1a Molecule	1.742
	B2M	Beta-2-Microglobulin	1.654
Innate Immune Receptor Signaling	NLRP3	NLR Family Pyrin Domain Containing 3	3.508
	NLRP5	NLR Family Pyrin Domain Containing 5	3.427
	TLR4	Toll Like Receptor 4	3.331
	NLRP14	NLR Family Pyrin Domain Containing 14	2.856
	NLRP10	NLR Family Pyrin Domain Containing 10	2.449
	BTK	Bruton Tyrosine Kinase	2.057
	NLRP12	NLR Family Pyrin Domain Containing 12	1.888
Cytokine Signaling	LTBP1	Latent Transforming Growth Factor Beta Binding Protein 1	1.918
	IL1RAPL1	Interleukin 1 Receptor Accessory Protein Like 1	1.805
	LTBP2	Latent Transforming Growth Factor Beta Binding Protein 2	1.690
	SERPINB4	Serpin Family B Member 4	2.325
Apoptosis/necrosis/Pyroptosis	BIRC7	Baculoviral IAP Repeat Containing 7	1.733
	CSMD3	CUB And Sushi Multiple Domains 3	5.517
Complement	CSMD1	CUB And Sushi Multiple Domains 1	4.948
	ITGAX	Integrin Subunit Alpha X	2.765
	C7	Complement C7	2.391
	MBL2	Mannose Binding Lectin 2	2.187
	FCN2	Ficolin 2	2.101
	MASP1	MBL Associated Serine Protease 1	1.952
	COLEC11	Collectin Subfamily Member 11	1.900
	CFHR4	Complement Factor H Related 4	1.758
	CFH	Complement Factor H	1.699

#### Mutations in driver genes with possible immune consequences

*TP53*, a multifunctional tumor suppressor mutated in over 50% of human cancers (Ozaki and Nakagawara, 2011), may regulate components of the innate and adaptive immune response (Hellmann et al., 2018; Shi and Jiang, 2021). TP53 mutations were found in 57% of WT patients and 35% of mKRAS patients. Mutations in liver kinase B1 (LKB1) gene, which encodes Serine/Threonine Kinase 11 (STK11), the 3^rd^ most mutated gene in the mKRAS cohort (after KRAS and TP53), regulates metabolism, energy sensing and modulates the stimulator of interferon genes (STING) pathway (Esteve-Puig et al., 2014; Kim et al., 2019). *STK11* mutations are associated with immunologically cold tumors (Schabath et al., 2016), increased neutrophil accumulation (Koyama et al., 2016), high expression of LAG3 ligand, FLG1 (Wang et al., 2019; Lehtiö et al., 2021), and poor outcomes in patients with mKRAS (Skoulidis et al., 2018). Anaplastic Lymphoma Kinase (*ALK*) mutations, found in 7.7% (24/313) of WT tumors, 4% (7/163) of mKRAS tumors, can generate an immunosuppressive microenvironment unresponsive to checkpoint blockade alone (Hellmann et al., 2018; Sankar et al., 2021).

#### Common over-mutated genes reflect previously identified pro-tumor pathways

Wild-type and mutant KRAS cohorts have many over-mutated genes in common (SR ≥ 1.65). Beta-2-Microglobulin (B2M) is a small gene (360bp) associated with resistance to checkpoint blockade. B2M was mutated in 2% of the wild-type cohort (6/313) and the mKRAS cohort (3/163) (WT SR = 1.653, mKRAS SR = 1.654). SERPINB4, (WT SR = 2.140, mKRAS SR = 2.325) is a serine protease inhibitor that inactivates granzyme M and promotes survival. Toll-like receptor 4 (TLR4) is commonly overexpressed in barrier epithelial cells and cancer (Luddy et al., 2014). TLR4 mutations were found in 12% (37/313) of the WT tumors and 11% (18/163) of the mKRAS tumors.

The *NLRP* family of receptors are under strong evolutionary selection in both cohorts (Figures 5A,B). *NLRP3* has the highest expression level and is over-mutated in both cohorts (WT SR = 3.150, mKRAS SR = 3.508). The *NLRP3* inflammasome alters the tumor microenvironment via secretion of pro-inflammatory cytokines IL1B (neutral in both cohorts) and IL18 (neutral in mKRAS, conserved in WT, SR = -1.144) (Kelley et al., 2019; Swanson et al., 2019). Similarly, the CD1 gene family is frequently mutated in both cohorts (Figures 3A,B). CD1E had the highest mutational prevalence (WT SR = 2.513, mKRAS SR = 2.329) and highest expression level (Figures 4A,B). Similar to ERAP1/2, CD1E and shapes the lipid antigen pool (Facciotti et al., 2011). CD1 molecules are mostly expressed by antigen presenting cells, however, aberrant expression of CD1 proteins by malignant cells can activate anti-tumor immune responses (Haraguchi et al., 2006; Hix et al., 2011).

## Discussion

The evolutionary arc of each tumor is likely unique with variations resulting from molecular properties of the initiating cells, heterogeneity in host responses, and stochastic variations in accumulated genetic events (Toki et al., 2018; Wang et al., 2021). However, prior theoretical studies have suggested commonalities should be found among tumors originating from the same tissue and/or initiation driver genes. Here, we demonstrate mKRAS and WT lung cancers generally exhibit convergent evolutionary selection among broad mechanisms of immune interactions and some gene families, but the specific genes selected, particularly those conserved, are frequently subtype specific. Such convergence of broad (phenotypic) properties through divergent molecular pathways is observed in nature. For example, cavefish develop a characteristic phenotype (no eyes or pigment) but through diverse genetic pathways depending on the genomic state of the founder population and stochastic perturbations (Gatenby et al., 2011).

Magalhães (Magalhães, 2021), in a PubMed analysis, found virtually every human gene has been associated with cancer. Within this vast data set, how can clinically important genes be selected? Here we propose a “Darwinian road map” can identify critical genes under evolutionary selection in clinical cancer cohorts. We examine gene conservation in immune related genes. Several of the genes identified in our studies have been noted previously. Pre-clinical and clinical work targeting many of these pathways are ongoing. The divergence of evolutionary selection in mKRAS and WT suggest pre-clinical experiments and clinical investigations with targeted drugs should be carefully designed with an understanding of the Darwinian importance of specific genes in specific tumor subtypes. For example, among immune modulators, common targets for immunotherapy (e.g., CD274, CTLA4) show neutral patterns of evolutionary selection. *PVR*, *PVRL2*, and *MICA*, which likely regulate NK cell function, are conserved in wild-type cohort. Nine anti-TIGIT (PVR and PVRL2 receptor) antibody therapies are currently being evaluated in 43 trials of advanced solid tumors, including NSCLC (Ge et al., 2021).

Each cohort conserves only one of two ERAP genes, which modify peptides for presentation by MHC class I. Clearly, the ERAP function is critical for optimal fitness in each cohort, and we note ERAP1/2 expression was detected in all 9,125 tumor specimens in The Cancer Genome Atlas database (TCGA), and deep deletions are rare (0.6–0.8%) (Compagnone et al., 2019. Targeting ERAP has been suggested by others and ERAP modulators are under development to modulate the anti-tumor immune response (Stratikos, 2014). However, our data raise an intriguing question: why do WT tumors highly conserve *ERAP1* while mKRAS tumors equally highly conserve *ERAP2*? *IRF3*, conserved in wild-type cohorts, regulates the expression of type I IFN genes and IFN stimulated genes (ISG) through binding interferon-stimulated response elements (ISRE) in their promoters. IRF3 is activated by several innate immune receptors (Figure 5F), including the cGAS/STING pathway, a novel immunotherapy target with ongoing clinical trials in cancer (Jiang et al., 2020).

Finally, in prior theoretical studies, computer simulations predicted therapies that disrupt conserved genes can have similar therapeutic efficacy to targeting driver genes (Gatenby et al., 2014). Thus, conserved genes in this study may be valuable clinical targets. Furthermore, simulations predicted combination therapy targeting a driver gene and a driver-specific conserved gene was highly lethal and often produced an evolutionary state in which no adaptive strategy was available, resulting in population extinction (Gatenby et al., 2014).

## Data Availability

The datasets presented in this study can be found in online repositories. The names of the repository/repositories and accession number(s) can be found below: mc3.v0.2.8.PUBLIC.maf.gz: https://gdc.cancer.gov/about-data/publications/mc3-2017. Genome Data Commons: https://portal.gdc.cancer.gov/repository.
