# Supplementary material for: Evolutionary selection identifies critical immune-relevant genes in lung cancer subtypes
Source: Front Genet. 2022 Aug 24;13:921447. doi: 10.3389/fgene.2022.921447 (PMC9451599; doi:10.3389/fgene.2022.921447)
Supplement: Supplementary file 1 [file DataSheet1.PDF]

Supplemental Figures

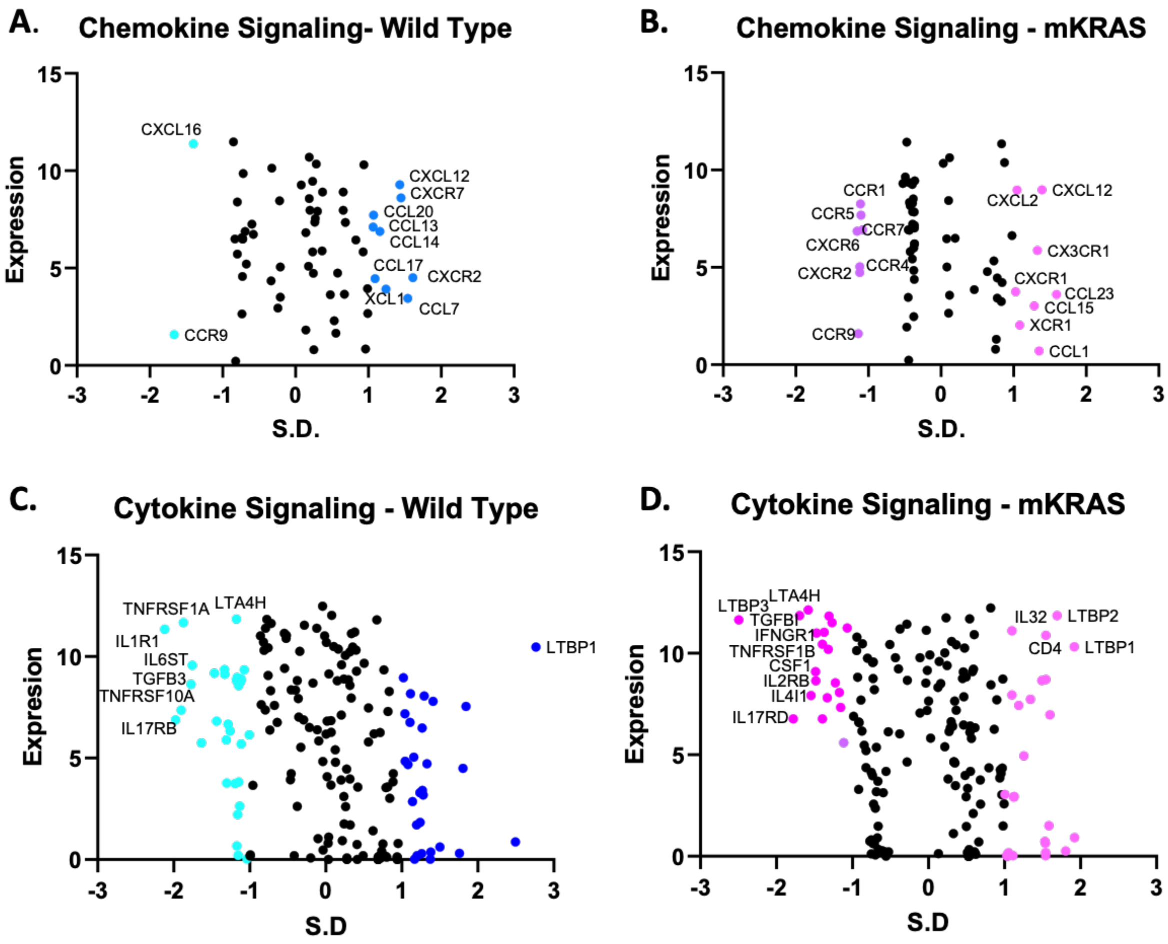

Supplemental Figure 1. Conservation profiles in chemokine genes (A-B) and cytokine genes (C-D). Gene conservation score, measure as the standard residual from the regression line (SR) graphed against genes expression levels (Log2) for WT (A and C) and mKRAS (B and D). Conserved genes (SR ≤ -1.0) and over-mutated genes (SR ≥ 1.0) are colored and labelled with text. (Standard residual (SR)

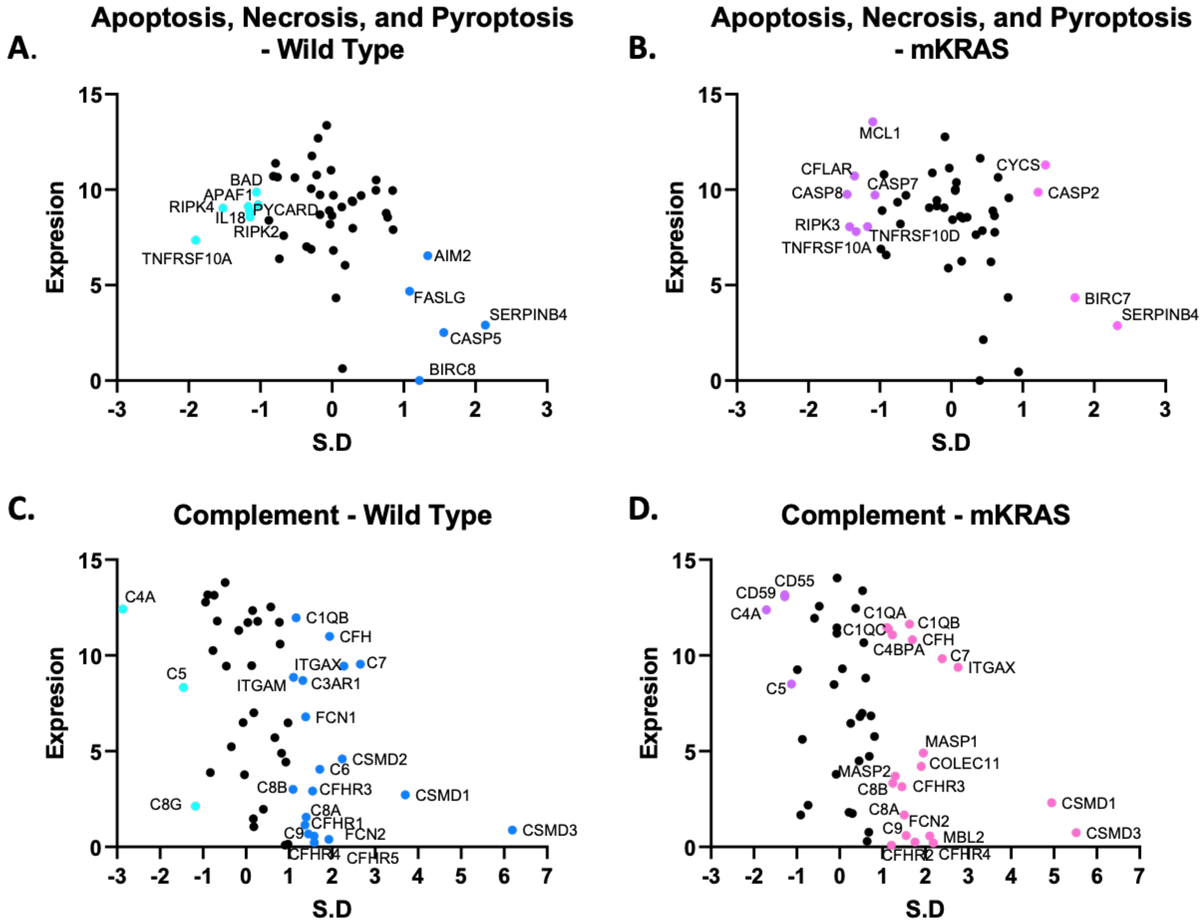

Supplemental Figure 2. Conservation profiles in cell death pathways (A-B) and complement system (C-D) genes. Gene conservation score, measure as the standard residual from the regression line (SR) graphed against genes expression levels (Log2) for WT (A) and mKRAS (B). Conserved genes (SR ≤ -1.0) and over-mutated genes (SR ≥ 1.0) are colored and labelled with text. Standard residual (SR).

Supplementary Tables

Process	Gene Symbols (Reference)
**Antigen processing and presentation**	B2M (1-5), CD1A (6-8), CD1B (6, 7), CD1C (6, 7), CD1D (6, 7), CD1E (6, 7) (9), CIITA (10, 11), ERAP1 (12-14), ERAP2 (12-14), HLA-A (1, 4)HLA-B (1, 4), HLA-C (1, 4), HLA-DMA (11), HLA-DMB (11), HLA-DOA (11), HLA-DOB (11), HLA-DPA1 (11), HLA-DPB1 (11), HLA-DQA1 (11), HLA-DQA2 (11), HLA-DQB1 (11), HLA-DQB2 (11), HLA-DRA (11), HLA-DRB1 (11), HLA-DRB5 (11), HLA-E (2), HLA-F (15), MEX3C (16), MTTP (6, 9), NLRC5 (4, 16), NPC1 (9, 17), NPC2 (9, 17), PSAP (9), PSMB8 (1) (18) (2, 16), PSMB9 (1, 18) (2, 16), RFX1 (19), RFX2 (19), RFX5 (10, 16), RFXANK (10, 16), TAP1 (1, 5, 18), TAPBP (1, 18)
**Immune Modulating Proteins**	ADAM17 (20), C10orf54, CCL2 (1), CD274 (1, 2) (5, 21), CD276 (1, 2) (5, 21), CD40 (21), CD47 (22), CD48 (23), CD80 (21), CD86 (21), CTLA4,ENTPD1 (24), FASLG (1), HCST, HLA-A (25), HLA-B (25), HLA-C (25), HLA-G (2), IDO1 (1, 20) (18), IDO2, LGALS1 (1), LGALS2 (1), LGALS3 (1), LGALS9 (1), MICA (2) (25), MICB (2, 26), NT5E (18, 20, 27), PDCD1LG2, PTGS1, PTGS2 (1), PVR (21, 26, 27), PVRIG (27), PVRL1 (26), PVRL2 (27, 28), PVRL3 (27, 28), PVRL4, RAET1E (2, 29), TGFB1 (1, 20, 30) (18), TNFRSF14 (27), TNFRSF6B, TNFSF4 (18, 21), TNFSF9 (21), ULBP1 (2, 29), ULBP2 (2, 29), ULBP3 (2, 29)
**Innate immune receptor signaling**	AGER (31), AIM2 (31), BTK (32), CASP1 (11, 33), CASP8 (31), CD14 (32), CIITA (11, 33), CTNNB1 (34), DDX3X, DDX41, DDX58 (11, 35), DHX58 (11, 35), DOK3, DOK4, DTX4, FADD (31),HMGB1 (31), IFI16 (31, 36), IFIH1 (11, 31), IL18 (33),

	IL1B (33), IRF3 (31, 32), IRF7 (31, 32), LRRFIP1, MAL (32), MAVS (11, 35), MB21D1 (35, 37), MyD88 (31), NLRC3 (11, 33), NLRC4 (11, 33), NLRC5 (11, 33), NLRP1 (11, 33), NLRP10 (11, 33), NLRP11 (11, 33), NLRP12 (11, 33), NLRP13 (11, 33), NLRP14 (11, 33), NLRP2 (11, 33), NLRP3 (11, 33), NLRP4 (11, 33), NLRP5 (11, 33), NLRP6 (11, 33), NLRP7 (11, 33), NLRP8 (11, 33), NLRP9 (11, 33), NLRX1 (11, 33, 38), NOD1 (11, 33), NOD2 (11, 33), OAS1 (11, 33), PYCARD (31), RIPK1 (31), TANK (32), TBK1 (35), TICAM1 (32), TLR1 (11, 32), TLR10 (11, 32), TLR2 (11, 32), TLR3 (11, 32), TLR4 (11, 32), TLR5 (11, 32), TLR6 (11, 32), TLR7 (11, 32), TLR8 (11, 32), TLR9 (11, 32), TMEM173 (37), TNIP1 (39), TRAF3 (32), TRAF6 (32), TRAM1 (32), TRIM56 (40), TUFM (38), ZBP1 (36)
**Interferon Signaling**	CREBBP, DOCK2, IFNA1, IFNA10, IFNA13, IFNA14, IFNA16, IFNA17, IFNA2, IFNA21, IFNA4, IFNA5, IFNA6, IFNA7, IFNA8, IFNAR1, IFNAR2, IFNB1, IFNE, IFNG, IFNGR1, IFNGR2, IL10RB, IL28A, IL28B, IL28RA, IL29, IRF1, IRF2, IRF2BP1, IRF2BP2, IRF2BPL, IRF3, IRF4, IRF5, IRF6, IRF7, IRF8, IRF9, JAK1, JAK2, JAK3, MAVS, PIAS1, PIAS2, PIAS3, PIAS4, PIK3CA, PTPN11, RAC1, RAC2, RAC3, SIRT1, SIRT2, SIRT3, SIRT4, SIRT5, SIRT7, SMARCA4, SOCS2, SOCS3, SOCS4, SOCS5, SOCS6, SOCS7, STAT1, STAT2, STAT3, STAT4, STAT5A, STAT5B, STAT6, TYK2, ULK1, USP18
**chemokine signaling**	CCL1, CCL11, CCL13 ,CCL14, CCL15, CCL16, CCL17, CCL18, CCL19, CCL2, CCL20, CCL21, CCL22, CCL23, CCL24, CCL25, CCL26, CCL2, CCL28, CCL3, CCL3L1, CCL4, CCL4L1, CCL4L2, CCL5, CCL7, CCL8, CCR1, CCR10, CCR2, CCR3, CCR4, CCR5, CCR6, CCR7, CCR8, CCR9, CCRL1, CX3CL1, CX3CR1, CXCL1, CXCL10, CXCL11,

	CXCL12, CXCL13, CXCL14, CXCL16, CXCL17, CXCL2, CXCL3, CXCL5,CXCL6, CXCL9, CXCR1, CXCR2, CXCR3, CXCR4, CXCR5, CXCR6, CXCR7, XCL1, XCL2, XCR1
**cytokine signaling**	CD27, CD4, CD40, CD40LG, CD70, CNTF, CSF1, CSF1R, CSF2, CSF2RA, CSF2RB, CSF3, CSF3R, Epo, EpoR, FAS, FASLG, FLT3, FLT3LG, IFNA1, IFNA10, IFNA13, IFNA14, IFNA16, IFNA17, IFNA2, IFNA21, IFNA4, IFNA5, IFNA6, IFNA7, IFNA8, IFNB1, IFNE, IFNG, IFNGR1, IFNGR2, IFNK, IFNW1, IL10, IL10RA, IL10RB, IL11RA, IL12A, IL12B, IL12RB1, IL12RB2, IL13, IL13RA1, IL13RA2, IL15, IL16, IL17A, IL17B, IL17C, IL17D, IL17F, IL17RA, IL17RB, IL17RC, IL17RD, IL17RE, IL17REL, IL18, IL18BP, IL18R1, IL18RAP, IL19, IL1A, IL1F10, IL1R1, IL1R2, IL1RAP, IL1RAPL1, IL1RAPL2, IL2, IL20, IL20RA, IL20RB, IL21, IL21R, IL22, IL23, IL23R, IL24, IL25, IL26, IL27, IL27RA, IL28A, IL28B, IL28RA, IL29, IL2RA, IL2RB, IL2RG, IL3, IL31, IL31RA, IL32, IL33, IL34, IL36A, IL36B, IL36G, IL36RN, IL37, IL3RA, IL4, IL4I1, IL4R, IL5, IL5RA, IL6, IL6R, IL6ST, IL7, IL7R, IL8, IL9, KIT, KITLG, LIF, LIFR, LTA, LTA4H, LTB, LTB4R, LTB4R2, LTBP1, LTBP2, LTBP3, LTBR, MPL, MST1, MST1R, NFKB1, OSM, OSMR, SPP1, TGFB1, TGFB1I1, TGFB2, TGFB3, TGFBI, TGFBR1, TGFBR2, TGFBR3, TNF, TNFRSF10A, TNFRSF10B, TNFRSF10C, TNFRSF10D, TNFRSF11A, TNFRSF11B,TNFRSF12A, TNFRSF13B, TNFRSF13C, TNFRSF14, TNFRSF17, TNFRSF19, TNFRSF1A, TNFRSF1B, TNFRSF4, TNFRSF6B, TNFRSF8, TNFRSF9, TNFSF10, TNFSF11, TNFSF12, TNFSF13B, TNFSF14, TNFSF18, TNFSF4, TNFSF8, TNFSF9, Tpo, TRAF3IP2, TSLP, TXLNA
**apoptosis, necrosis, and pyroptosis**	Aim2 (41), APAF1, BAD (42), Bcl-2 (2) (43) (44), BCL2L1 (2), BIRC5 (45) (46),

	BIRC6, BIRC7, BIRC8, CASP1 (41), CASP10, CASP14, CASP2 (42), CASP3, CASP4 (41), CASP5 (41), CASP6 (42), CASP7, CASP8 (42), CASP8AP2, CASP9, CFLAR (47) (48) CYCS, CYLD, FADD (1), FAS (1) (49), FASLG, GSDMD (41), IL18 (41), IL1B (41), MCL1 (42), Nlrc4 (41), NLRP1 (41), PYCARD (31), RIPK1 (41), RIPK2, RIPK3 (41), RIPK4, SERPINB4 (50), SERPINB9 (51) (52) (53) {topfer2011}, STAT3 (2), TNFRSF10A (1), TNFRSF10B (1), TNFRSF10C (1), TNFRSF10D (1), TNFRSF11B (1), TNFRSF6B (1) (2), TNFSF10 (1)
**Complement**	C1QA, C1QB, C1QC, C1S, C2, C3, C3AR1, C4A, C4B, C4BPA, C4BPB, C5, C6, C7, C8A, C8B, C8G, C9, CD46, CD55, CD59, CD93, CFB, CFDP1, CFH, CFHR1, CFHR2, CFHR3, CFHR4, CFHR5, CFP, COLEC10, COLEC11, CR1, CR2, CSMD1, CSMD2, CSMD3, ELANE, F2, FCN1, FCN2, FCN3, ITGAM, ITGAX, ITGB2, MASP1, MASP2, MBL2, SERPING1, VSIG4, VTN

Supplemental Table 1. Gene lists for broad categories of tumor-immune interactions.

	Mutant KRAS	Wild Type
Average # of gene mutations/tumor	300	348
# of conserved genes (SR ≤ -1.0)	3,011	2,587
# of highly conserved genes (SR ≤ -2.0)	160	248
# of hypermutated genes (SR ≥ 1.0)	2,581	2,398
# of highly hypermutated genes (SR ≥ 2.0)	518	593
# of frequently mutated genes (≥10% of patients)	157	208
# of genes mutated in 0 patients	5,621	2,323

Supplemental Table 2. The number of total genes conserved or hypermutated varied between mutant KRAS and wild-type lung tumours. Mutation rates of 17,615 genes were calculated in mutant KRAS (n= 163 patients) and wild-type (n=313 patients) lung tumours. Numbers of conserved or hypermutated genes out of 17,615 genes examined. Genes with an SR ≥ 1.0 are hypermutated and genes with an SR ≤ -1.0 are conserved genes (under mutated). Others have an expected number of mutations (Background mutations). Standard residual (SR).

	Gene	mutated samples	total samples	SR score
**Wild-type**	TP53	177	313	11.94
	CSMD3	135	313	6.19
	KEAP1	59	313	6.07
	RYR2	125	313	5.58
	STK11	27	313	5.52
	ZFHX4	104	313	5.49
	COL11A1	82	313	5.44
	ZNF536	69	313	5.42
	CDH10	59	313	5.35
	NRXN1	65	313	5.07
**mKRAS**	KRAS	163	163	15.14
	TP53	57	163	8.18

STK11	32	163	7.72
ZFHX4	60	163	5.83
KEAP1	31	163	5.64
CSMD3	65	163	5.52
RYR2	66	163	5.44
ZNF536	37	163	5.22
LRP1B	66	163	5.04
CSMD1	46	163	4.95

Supplemental Table 3. Genes with the highest mutation score for mutant KRAS and wild-type cohorts. Non-synonymous mutation rates of 17,615 genes were calculated in mutant KRAS (n= 163 patients) and wild-type (n=313 patients) lung tumours. Standard residual (SR).

References

1. Töpfer K, Kempe S, Müller N, Schmitz M, Bachmann M, Cartellieri M, Schackert G, Temme A. Tumor evasion from T cell surveillance. J Biomed Biotechnol. 2011;2011:918471. Epub 2011/11/15. doi: 10.1155/2011/918471. PubMed PMID: 22190859; PMCID: PMC3228689.

2. Ting Koh Y, Luz García-Hernández M, Martin Kast W. Tumor Immune Escape Mechanisms. In: Teicher BA, editor. Cancer Drug Resistance. Totowa, NJ: Humana Press; 2006. p. 577-602.

3. O'Donnell JS, Smyth MJ, Teng MW. Acquired resistance to anti-PD1 therapy: checkmate to checkpoint blockade? Genome Med. 2016;8(1):111. doi: 10.1186/s13073-016-0365-1. PubMed PMID: 27782862; PMCID: PMC5080691.

4. Ozcan M, Janikovits J, von Knebel Doeberitz M, Kloor M. Complex pattern of immune evasion in MSI colorectal cancer. Oncoimmunology. 2018;7(7):e1445453. doi: 10.1080/2162402X.2018.1445453. PubMed PMID: 29900056; PMCID: PMC5993484.

5. Wang Q, Wu X. Primary and acquired resistance to PD-1/PD-L1 blockade in cancer treatment. Int Immunopharmacol. 2017;46:210-9. Epub 2017/03/18. doi: 10.1016/j.intimp.2017.03.015. PubMed PMID: 28324831.

6. Barral DC, Brenner MB. CD1 antigen presentation: how it works. Nat Rev Immunol. 2007;7(12):929-41. Epub 2007/11/27. doi: 10.1038/nri2191. PubMed PMID: 18037897.

7. Van Kaer L, Wu L, Joyce S. Mechanisms and Consequences of Antigen Presentation by CD1. Trends Immunol. 2016;37(11):738-54. Epub 2016/09/14. doi: 10.1016/j.it.2016.08.011. PubMed PMID: 27623113; PMCID: PMC5159200.

8. Coventry B, Heinzel S. CD1a in human cancers: a new role for an old molecule. Trends Immunol. 2004;25(5):242-8. Epub 2004/04/22. doi: 10.1016/j.it.2004.03.002. PubMed PMID: 15099564.

9. Teyton L. Role of lipid transfer proteins in loading CD1 antigen-presenting molecules. J Lipid Res. 2018;59(8):1367-73. Epub 2018/03/22. doi: 10.1194/jlr.R083212. PubMed PMID: 29559523; PMCID: PMC6071766.

10. Kloor M, Michel S, von Knebel Doeberitz M. Immune evasion of microsatellite unstable colorectal cancers. Int J Cancer. 2010;127(5):1001-10. Epub 2010/03/04. doi: 10.1002/ijc.25283. PubMed PMID: 20198617.

11. Wosen JE, Mukhopadhyay D, Macaubas C, Mellins ED. Epithelial MHC Class II Expression and Its Role in Antigen Presentation in the Gastrointestinal and Respiratory Tracts. Front Immunol. 2018;9:2144. Epub 2018/10/16. doi: 10.3389/fimmu.2018.02144. PubMed PMID: 30319613; PMCID: PMC6167424.

12. Nagarajan NA, Gonzalez F, Shastri N. Nonclassical MHC class Ib-restricted cytotoxic T cells monitor antigen processing in the endoplasmic reticulum. Nat Immunol. 2012;13(6):579-86. Epub 2012/04/24. doi: 10.1038/ni.2282. PubMed PMID: 22522492; PMCID: PMC3362685.

13. Yewdell JW, Lu X. Don't mess with ERAAP! Nat Immunol. 2012;13(6):526-8. Epub 2012/05/23. doi: 10.1038/ni.2306. PubMed PMID: 22610241; PMCID: PMC3777675.

14. Saulle I, Vicentini C, Clerici M, Biasin M. An Overview on ERAP Roles in Infectious Diseases. Cells. 2020;9(3). Epub 2020/03/19. doi: 10.3390/cells9030720. PubMed PMID: 32183384; PMCID: PMC7140696.

15. Lin A, Yan W-H. The Emerging Roles of Human Leukocyte Antigen-F in Immune Modulation and Viral Infection. Frontiers in Immunology. 2019;10(964). doi: 10.3389/fimmu.2019.00964.

16. Jongsma MLM, Neefjes J, Spaapen RM. Playing hide and seek: Tumor cells in control of MHC class I antigen presentation. Mol Immunol. 2021;136:36-44. Epub 2021/06/04. doi: 10.1016/j.molimm.2021.05.009. PubMed PMID: 34082257.

17. Schrantz N, Sagiv Y, Liu Y, Savage PB, Bendelac A, Teyton L. The Niemann-Pick type C2 protein loads isoglobotrihexosylceramide onto CD1d molecules and contributes to the thymic selection of NKT cells. J Exp Med. 2007;204(4):841-52. Epub 2007/03/29. doi: 10.1084/jem.20061562. PubMed PMID: 17389239; PMCID: PMC2118543.

18. Vinay DS, Ryan EP, Pawelec G, Talib WH, Stagg J, Elkord E, Lichtor T, Decker WK, Whelan RL, Kumara HM, Signori E, Honoki K, Georgakilas AG, Amin A, Helferich WG, Boosani CS, Guha G, Ciriolo MR, Chen S, Mohammed SI, Azmi AS, Keith WN, Bilsland A, Bhakta D, Halicka D, Fujii H, Aquilano K, Ashraf SS, Nowsheen S, Yang X, Choi BK, Kwon BS. Immune evasion in cancer: Mechanistic basis and therapeutic strategies. Semin Cancer Biol. 2015;35 Suppl:S185-98. Epub 2015/03/25. doi: 10.1016/j.semcancer.2015.03.004. PubMed PMID: 25818339.

19. Pugliatti L, Derre J, Berger R, Ucla C, Reith W, Mach B. The genes for MHC class II regulatory factors RFX1 and RFX2 are located on the short arm of chromosome 19. Genomics. 1992;13(4):1307-10. Epub 1992/08/01. doi: 10.1016/0888-7543(92)90052-t. PubMed PMID: 1505960.

20. Guillerey C, Huntington ND, Smyth MJ. Targeting natural killer cells in cancer immunotherapy. Nat Immunol. 2016;17(9):1025-36. doi: 10.1038/ni.3518. PubMed PMID: 27540992.

21. Wykes MN, Lewin SR. Immune checkpoint blockade in infectious diseases. Nat Rev Immunol. 2018;18(2):91-104. Epub 2017/10/11. doi: 10.1038/nri.2017.112. PubMed PMID: 28990586; PMCID: PMC5991909.

22. Willingham SB, Volkmer JP, Gentles AJ, Sahoo D, Dalerba P, Mitra SS, Wang J, Contreras-Trujillo H, Martin R, Cohen JD, Lovelace P, Scheeren FA, Chao MP, Weiskopf K, Tang C, Volkmer AK, Naik TJ, Storm

TA, Mosley AR, Edris B, Schmid SM, Sun CK, Chua MS, Murillo O, Rajendran P, Cha AC, Chin RK, Kim D, Adorno M, Raveh T, Tseng D, Jaiswal S, Enger PO, Steinberg GK, Li G, So SK, Majeti R, Harsh GR, van de Rijn M, Teng NN, Sunwoo JB, Alizadeh AA, Clarke MF, Weissman IL. The CD47-signal regulatory protein alpha (SIRPa) interaction is a therapeutic target for human solid tumors. Proc Natl Acad Sci U S A. 2012;109(17):6662-7. Epub 2012/03/28. doi: 10.1073/pnas.1121623109. PubMed PMID: 22451913; PMCID: PMC3340046.

23. Chester C, Fritsch K, Kohrt HE. Natural Killer Cell Immunomodulation: Targeting Activating, Inhibitory, and Co-stimulatory Receptor Signaling for Cancer Immunotherapy. Front Immunol. 2015;6:601. Epub 2015/12/24. doi: 10.3389/fimmu.2015.00601. PubMed PMID: 26697006; PMCID: PMC4667030.

24. Guillerey C, Smyth MJ. NK Cells and Cancer Immunoediting. Curr Top Microbiol Immunol. 2016;395:115-45. doi: 10.1007/82_2015_446. PubMed PMID: 26025472.

25. Pancione M, Giordano G, Remo A, Febbraro A, Sabatino L, Manfrin E, Ceccarelli M, Colantuoni V. Immune escape mechanisms in colorectal cancer pathogenesis and liver metastasis. J Immunol Res. 2014;2014:686879. Epub 2014/01/16. doi: 10.1155/2014/686879. PubMed PMID: 24741617; PMCID: PMC3987978.

26. Chan CJ, Smyth MJ, Martinet L. Molecular mechanisms of natural killer cell activation in response to cellular stress. Cell Death Differ. 2014;21(1):5-14. Epub 2013/04/13. doi: 10.1038/cdd.2013.26. PubMed PMID: 23579243; PMCID: PMC3857624.

27. Stamm H, Wellbrock J, Fiedler W. Interaction of PVR/PVRL2 with TIGIT/DNAM-1 as a novel immune checkpoint axis and therapeutic target in cancer. Mamm Genome. 2018. Epub 2018/08/23. doi: 10.1007/s00335-018-9770-7. PubMed PMID: 30132062.

28. Chan CJ, Martinet L, Gilfillan S, Souza-Fonseca-Guimaraes F, Chow MT, Town L, Ritchie DS, Colonna M, Andrews DM, Smyth MJ. The receptors CD96 and CD226 oppose each other in the regulation of natural killer cell functions. Nat Immunol. 2014;15(5):431-8. Epub 2014/03/25. doi: 10.1038/ni.2850. PubMed PMID: 24658051.

29. McGilvray RW, Eagle RA, Watson NF, Al-Attar A, Ball G, Jafferji I, Trowsdale J, Durrant LG. NKG2D ligand expression in human colorectal cancer reveals associations with prognosis and evidence for immunoediting. Clin Cancer Res. 2009;15(22):6993-7002. doi: 10.1158/1078-0432.CCR-09-0991. PubMed PMID: 19861434; PMCID: PMC2778653.

30. Gao Y, Souza-Fonseca-Guimaraes F, Bald T, Ng SS, Young A, Ngiow SF, Rautela J, Straube J, Waddell N, Blake SJ, Yan J, Bartholin L, Lee JS, Vivier E, Takeda K, Messaoudene M, Zitvogel L, Teng MWL, Belz GT, Engwerda CR, Huntington ND, Nakamura K, Holzel M, Smyth MJ. Tumor immunoevasion by the conversion of effector NK cells into type 1 innate lymphoid cells. Nat Immunol. 2017;18(9):1004-15. doi: 10.1038/ni.3800. PubMed PMID: 28759001.

31. Amarante-Mendes GP, Adjemian S, Branco LM, Zanetti LC, Weinlich R, Bortoluci KR. Pattern Recognition Receptors and the Host Cell Death Molecular Machinery. Front Immunol. 2018;9:2379. Epub 2018/11/22. doi: 10.3389/fimmu.2018.02379. PubMed PMID: 30459758; PMCID: PMC6232773.

32. Kawasaki T, Kawai T. Toll-Like Receptor Signaling Pathways. Frontiers in Immunology. 2014;5(461). doi: 10.3389/fimmu.2014.00461.

33. Velloso FJ, Trombetta-Lima M, Anschau V, Sogayar MC, Correa RG. NOD-like receptors: major players (and targets) in the interface between innate immunity and cancer. Biosci Rep. 2019;39(4). Epub 2019/03/07. doi: 10.1042/BSR20181709. PubMed PMID: 30837326; PMCID: PMC6454022.

34. You H, Lin Y, Lin F, Yang M, Li J, Zhang R, Huang Z, Shen Q, Tang R, Zheng C. beta-Catenin Is Required for the cGAS/STING Signaling Pathway but Antagonized by the Herpes Simplex Virus 1 US3 Protein. J Virol. 2020;94(5). Epub 2019/12/06. doi: 10.1128/JVI.01847-19. PubMed PMID: 31801859; PMCID: PMC7022340.

35. Elion DL, Cook RS. Harnessing RIG-I and intrinsic immunity in the tumor microenvironment for therapeutic cancer treatment. Oncotarget. 2018;9(48):29007-17. Epub 2018/07/11. doi: 10.18632/oncotarget.25626. PubMed PMID: 29989043; PMCID: PMC6034747.

36. Upton JW, Kaiser WJ, Mocarski ES. DAI/ZBP1/DLM-1 complexes with RIP3 to mediate virus-induced programmed necrosis that is targeted by murine cytomegalovirus vIRA. Cell Host Microbe. 2012;11(3):290-7. Epub 2012/03/20. doi: 10.1016/j.chom.2012.01.016. PubMed PMID: 22423968; PMCID: PMC3531981.

37. Ding W, Ma Y, Zhu W, Pu W, Zhang J, Qian F, Zhou Y, Deng Y, Guo S, Wang J, Zhou X. MICA (*)012:01 Allele Facilitates the Metastasis of KRAS-Mutant Colorectal Cancer. Front Genet. 2020;11:511. Epub 2020/06/13. doi: 10.3389/fgene.2020.00511. PubMed PMID: 32528529; PMCID: PMC7264413.

38. Lei Y, Wen H, Yu Y, Taxman DJ, Zhang L, Widman DG, Swanson KV, Wen KW, Damania B, Moore CB, Giguère PM, Siderovski DP, Hiscott J, Razani B, Semenkovich CF, Chen X, Ting JP. The mitochondrial proteins NLRX1 and TUFM form a complex that regulates type I interferon and autophagy. Immunity. 2012;36(6):933-46. Epub 2012/07/04. doi: 10.1016/j.immuni.2012.03.025. PubMed PMID: 22749352; PMCID: PMC3397828.

39. Mirza N, Sowa AS, Lautz K, Kufer TA. NLRP10 Affects the Stability of Abin-1 To Control Inflammatory Responses. The Journal of Immunology. 2019;202(1):218-27. doi: 10.4049/jimmunol.1800334.

40. Shen Y, Li NL, Wang J, Liu B, Lester S, Li K. TRIM56 is an essential component of the TLR3 antiviral signaling pathway. J Biol Chem. 2012;287(43):36404-13. Epub 2012/09/06. doi: 10.1074/jbc.M112.397075. PubMed PMID: 22948160; PMCID: PMC3476306.

41. Man SM, Karki R, Kanneganti TD. Molecular mechanisms and functions of pyroptosis, inflammatory caspases and inflammasomes in infectious diseases. Immunol Rev. 2017;277(1):61-75. Epub 2017/05/04. doi: 10.1111/imr.12534. PubMed PMID: 28462526; PMCID: PMC5416822.

42. Fernald K, Kurokawa M. Evading apoptosis in cancer. Trends Cell Biol. 2013;23(12):620-33. Epub 2013/08/16. doi: 10.1016/j.tcb.2013.07.006. PubMed PMID: 23958396; PMCID: PMC4091735.

43. Muenst S, Läubli H, Soysal SD, Zippelius A, Tzankov A, Hoeller S. The immune system and cancer evasion strategies: therapeutic concepts. J Intern Med. 2016;279(6):541-62. doi: 10.1111/joim.12470. PubMed PMID: 26748421.

44. Stewart TJ, Abrams SI. How tumours escape mass destruction. Oncogene. 2008;27(45):5894-903. doi: 10.1038/onc.2008.268. PubMed PMID: 18836470.

45. Garg H, Suri P, Gupta JC, Talwar GP, Dubey S. Survivin: a unique target for tumor therapy. Cancer Cell Int. 2016;16:49. Epub 2016/06/23. doi: 10.1186/s12935-016-0326-1. PubMed PMID: 27340370; PMCID: PMC4917988.

46. Ji W, Chen J, Mi Y, Wang G, Xu X, Wang W. Role of natural killer cells in liver transplantation treatment of liver cancer. Exp Ther Med. 2017;14(3):2380-4. Epub 2017/10/01. doi: 10.3892/etm.2017.4748. PubMed PMID: 28962171; PMCID: PMC5609091.

47. Carnevale G, Carpino G, Cardinale V, Pisciotta A, Riccio M, Bertoni L, Gibellini L, De Biasi S, Nevi L, Costantini D, Overi D, Cossarizza A, de Pol A, Gaudio E, Alvaro D. Activation of Fas/FasL pathway and the role of c-FLIP in primary culture of human cholangiocarcinoma cells. Sci Rep. 2017;7(1):14419. Epub 2017/10/31. doi: 10.1038/s41598-017-14838-3. PubMed PMID: 29089545; PMCID: PMC5663931.

48. Medema JP, de Jong J, van Hall T, Melief CJ, Offringa R. Immune escape of tumors in vivo by expression of cellular FLICE-inhibitory protein. J Exp Med. 1999;190(7):1033-8. PubMed PMID: 10510093; PMCID: PMC2195655.

49. Wang M, Zhang C, Song Y, Wang Z, Wang Y, Luo F, Xu Y, Zhao Y, Wu Z, Xu Y. Mechanism of immune evasion in breast cancer. Onco Targets Ther. 2017;10:1561-73. doi: 10.2147/OTT.S126424. PubMed PMID: 28352189; PMCID: PMC5359138.

50. de Koning PJ, Kummer JA, de Poot SA, Quadir R, Broekhuizen R, McGettrick AF, Higgins WJ, Devreese B, Worrall DM, Bovenschen N. Intracellular serine protease inhibitor SERPINB4 inhibits granzyme M-induced cell death. PLoS One. 2011;6(8):e22645. Epub 2011/08/03. doi: 10.1371/journal.pone.0022645. PubMed PMID: 21857942; PMCID: PMC3152296.

51. Bird CH, Sutton VR, Sun J, Hirst CE, Novak A, Kumar S, Trapani JA, Bird PI. Selective regulation of apoptosis: the cytotoxic lymphocyte serpin proteinase inhibitor 9 protects against granzyme B-mediated apoptosis without perturbing the Fas cell death pathway. Mol Cell Biol. 1998;18(11):6387-98. PubMed PMID: 9774654; PMCID: PMC109224.

52. Medema JP, de Jong J, Peltenburg LT, Verdegaal EM, Gorter A, Bres SA, Franken KL, Hahne M, Albar JP, Melief CJ, Offringa R. Blockade of the granzyme B/perforin pathway through overexpression of the serine protease inhibitor PI-9/SPI-6 constitutes a mechanism for immune escape by tumors. Proc Natl Acad Sci U S A. 2001;98(20):11515-20. Epub 2001/09/20. doi: 10.1073/pnas.201398198. PubMed PMID: 11562487; PMCID: PMC58761.

53. Kaiserman D, Bird PI. Control of granzymes by serpins. Cell Death Differ. 2010;17(4):586-95. Epub 2009/11/06. doi: 10.1038/cdd.2009.169. PubMed PMID: 19893573.
